# Extreme Environment Effects on Cognitive Functions: A Longitudinal Study in High Altitude in Antarctica

**DOI:** 10.3389/fnhum.2016.00331

**Published:** 2016-06-30

**Authors:** Irén Barkaszi, Endre Takács, István Czigler, László Balázs

**Affiliations:** ^1^Environmental Adaptation and Space Research Group, Institute of Cognitive Neuroscience and Psychology, Research Centre for Natural Sciences, Hungarian Academy of SciencesBudapest, Hungary; ^2^Department of Experimental Psychology, Institute of Psychology, Debrecen UniversityDebrecen, Hungary; ^3^Faculty of Education and Psychology, Eötvös Loránd UniversityBudapest, Hungary

**Keywords:** event-related potentials (ERP), cognitive functions, extreme environment, Antarctica, long-term isolation, hypoxia, sunshine duration, successful adaptation

## Abstract

This paper focuses on the impact of long-term Antarctic conditions on cognitive processes. Behavioral responses and event-related potentials were recorded during an auditory distraction task and an attention network paradigm. Participants were members of the over-wintering crew at Concordia Antarctic Research Station. Due to the reduced partial pressure of oxygen this environment caused moderate hypoxia. Beyond the hypoxia, the fluctuation of sunshine duration, isolation and confinement were the main stress factors of this environment. We compared 6 measurement periods completed during the campaign. Behavioral responses and N1/MMN (mismatch negativity), N1, N2, P3, RON (reorientation negativity) event-related potential components have been analyzed. Reaction time decreased in both tasks in response to repeated testing during the course of mission. The alerting effect increased, the inhibition effect decreased and the orienting effect did not change in the ANT task. Contrary to our expectations the N2, P3, RON components related to the attentional functions did not show any significant changes. Changes attributable to early stages of information processing were observed in the ANT task (N1 component) but not in the distraction task (N1/MMN). The reaction time decrements and the N1 amplitude reduction in ANT task could be attributed to sustained effect of practice. We conclude that the Antarctic conditions had no negative impacts on cognitive activity despite the presence of numerous stressors.

## Introduction

The aim of this study was to investigate cognitive activity in Antarctic conditions. On this end we introduced an auditory distraction task (Schroger and Wolff, 1998), and the attention network paradigm (Fan et al., 2009). Beside the behavioral data in both tasks event-related brain potentials (ERPs) were measured. We compared 6 measurements (so-called cycles) that were completed during the campaign.

Long-duration missions in hostile, isolated and confined environment involve many stressors that affect crew's performance and cognitive functions. The decrement in performance and cognitive functions might bring about serious problems, even fatal human errors, especially during space missions. Therefore, it is necessary to investigate the possibility of cognitive changes in conditions with some similarities (e.g., extreme environment, isolation) to the conditions of spaceflight.

Participants of the present study were members of the over-wintering crew at Concordia Antarctic Research Station, which is a joint French-Italian research facility. The station is located on the Antarctic Plateau 3233 m above sea level. It is one of the coldest places on Earth, in 2011 the average air temperature was −51.2°C (min. −76.4°C, max. −19°C). The air temperature of that year was −35.8°C in summer and −64.4°C in winter. The sun disappears completely during winter (from 2–3 of May to 9–10 of August). The over-wintering crewmembers (13 participants) had to adapt to this harsh environment. The main stressors of this environment are hypoxia, fluctuation of sunshine duration, isolation and confinement.

At the Concordia station (3233 m altitude) the average air pressure was 482.5 Hgmm between February and December 2011 (this value corresponds to 3880 m along the Equator). Due to the reduced partial pressure of oxygen, this condition caused moderate *hypoxia*. The average nocturnal oxygen saturation (Sp_O2_) measured by pulse-oximetry was 85.1–87.9% during the campaign (Tellez et al., 2014). Our data on diurnal Sp_O2_ exhibits slightly higher values, 89–94% during the stay, still noticeably under the normal Sp_O2_of 96–100% (see exact numbers in Results). Additionally, Tellez et al. (2014) reported, for most of the participants, clinically severe level of periodic breathing all through the year-long campaign without any improvement. The heart rate, frequency of respiration and length of periods with periodic breathing remained stable during the campaign. Another study (Abeln et al., 2015) investigated the cognitive performance and mood in the same crewmembers in 2011. They separated subjects into active and inactive groups based on their training load during the campaign. Subjects did not show changes in cognitive performance during their stay. Concerning the mood state, they measured the physical well-being and the perceived psychological state (perceived psychological strain and perceived motivational state). The deterioration of mood was observed in the inactive group which means that they felt physically worse, their motivation decreased and they became more strained. The mood of the active group remained stable. They also registered eyes closed resting EEG. Alpha and beta bands decreased in the active group during the campaign and alpha band increased in the second session and remained in this level in the inactive group during their stay.

In this range of altitude (3880 +/− 500 m) compromised psychomotor functions, arithmetical skills (Shukitt-Hale et al., 1998), impairments in short-term memory (Bartholomew et al., 1999), multi-task performance (Adam et al., 2008), logical reasoning (Green and Morgan, 1985) have been observed in response to acute exposure. Long-term sojourn in high altitude locations affected long-latency event-related potentials in several studies (Singh et al., 2004; Thakur et al., 2011; Ma et al., 2015).

Besides the direct effect of systemic hypoxaemia on brain, hypoxia might impact waking neurocognitive functions through the deterioration of sleep quality. Sleep in high altitude becomes more superficial with frequent awakenings. The proportion of REM sleep and slow-wave sleep (deep sleep; stages 3 and 4) decreases and the proportion of light sleep (stages 1 and 2) increases (Wickramasinghe and Anholm, 1999). The occurrence of periodic breathing during the campaign in question (Tellez et al., 2014) may indicate deteriorated sleep quality. However, the relationship of periodic breathing with sleep fragmentation and decrease of slow-wave sleep is controversial (Johnson et al., 2010; Nussbaumer-Ochsner et al., 2012) and lacking data on chronic exposures. Still, Stadelmann et al. (2014) suggest that central apneas during periodic breathing initiate previously undetected sleep microarousals. Sleep fragmentation in turn might lead to daytime sleepiness, vigilance decrement and cognitive impairments (Bonnet and Arand, 2003).

Accordingly, Collet et al. (2015) observed compromised sleep quality in a different cohort of subjects at Concordia station. Total sleep time was shorter, sleep efficiency was lower and wake periods after sleep onset were longer at Concordia station compared to sea level station Dumont d'Urville, highlighting the role of hypoxia in sleep disruption.

Beyond hypoxia, the fluctuation of sunshine duration is another main stressor in this environment. Collet et al. (2015) found that constant *sunlight* exposure during summer has initiated sleep fragmentation at both high-altitude and sea-level stations. Moreover, the disappearance of sun for the winter triggers affective problems in some subjects (Palinkas and Suedfeld, 2008).

As for other stressors of this environment, crewmembers stay in complete physical *isolation* and *confinement* during the winter with minimal or no chance of evacuation in emergency. Isolation and confinement are traditionally thought to impact mood, motivation, and interpersonal relations (Palinkas and Suedfeld, 2008) so they might exert influence on cognition and attention through these factors, although exact pathomechanisms are little-known.

Based on the negative effects of stressors, we expected deterioration of cognitive functions mainly during the winter (third and fourth measurements) because of several factors. First, the sun disappeared completely in these measurement periods which might exacerbate sleep problems (Bhargava et al., 2000) and might trigger affective problems. Second, crewmembers had already spent a few months in Antarctica and according to Reed et al. (2001) 3 months residence in Antarctica had detrimental effect on cognition. Furthermore, few months of the mission in isolation and confinement have passed and the end of the mission is too far away that might also increase emotional and interpersonal problems (so called third-quarter phenomenon; Bechtel and Berning, 1991) in the fourth measurement.

However, due to successful coping with stress, a polar expedition may induce also positive (so-called salutogenic, Antonovsky, 1987) effects, like enhanced self-efficiency and personal growth (Palinkas et al., 2000; Palinkas and Suedfeld, 2008) that may counteract the detrimental effects of stressors.

The results of cognitive performance investigations on Antarctica are controversial. Some researchers reported decreased performance (Reed et al., 2001) while others did not report any cognitive change (Le Scanff et al., 1997) and still others even measured improvement (Defayolle et al., 1985). To settle the controversy, in our Antarctic study, participants had to perform two cognitive tasks while brain electrical activity (EEG) had been recorded. Occasionally event-related brain potentials (ERP) might be more sensitive to slight changes of brain activity than behavioral measures (Czigler et al., 1994). We hypothesized that fronto-parietal attentional functions become impaired in Antarctica as these abilities are particularly sensitive to stressors, such as *hypoxia* (Virués-Ortega et al., 2004), *mental fatigue* (Lorist et al., 2000), *sleep loss* (Jones and Harrison, 2001), and sleep quality (Schapkin et al., 2006), therefore we selected tasks capable to assess these functions.

The auditory distraction paradigm (Schroger and Wolff, 1998) consists of a duration-discrimination task with infrequent, task-irrelevant changes. Typical findings regarding the irrelevant changes include prolonged reaction time and higher error rate accompanied by a series of ERP components: early negativity (N1/MMN), P3a and reorienting negativity (RON; Horváth et al., 2008). N1/MMN reflects the pre-attentive automatic detection of change (Näätänen et al., 2007) and usually peaks between 100 and 250 ms from the onset of change. P3a reflects attentional orientation toward the detected change and exhibits a fronto-central scalp distribution with a peak stimulus latency of 280–500 ms. RON is hypothesized to signal the subsequent reorientation of attention to the relevant information. This component exhibits a fronto-central scalp distribution with a peak stimulus latency of ~500–600 ms after the onset of change (Schroger and Wolff, 1998).

The Attention Network Test (ANT) combines cued detection task (Posner, 1980) with a flanker-type paradigm (Eriksen and Eriksen, 1974) and the effect of warning signal. The attention system comprises three anatomically and functionally separate networks (alerting, orienting and executive control functions; Posner and Petersen, 1990; Fan et al., 2002) and these networks are calculated as reaction time differences of specified task conditions. The alerting network is responsible for a state of arousal. The orienting network is involved in the selection of information among multiple sensory inputs or locations, while executive control involves a set of operations including detecting and resolving conflicts (Fan et al., 2009). Only few studies investigated the event-related potentials (ERPs) in the ANT task (Neuhaus et al., 2010). We expected changes in those ERP components which are related to the frontal attentional functions and that are known to be influenced by stressors described above. Such components are the N2 and the late positivity (P3 complex).

*N2 component*. The peak latency of the N2 component is about 200 ms after the stimulus onset (Courchesne et al., 1975; Squires et al., 1975; Näätänen and Picton, 1986). This component is interpreted as a correlate of response inhibition or conflict detection (Kok, 1986; Kiefer et al., 1998; Donkers and van Boxtel, 2004; Folstein and Van Petten, 2008; Smith et al., 2010). In a study by Kida (1997), N2 latency increased as a result of *hypoxia*, but the study of Wesensten et al. (1993) did not find effects of hypoxia on N2. Ma et al. (2015) found delayed latency of no-go N2 and larger go and no-go N2 amplitude in the high-altitude group compared to the low-altitude group. N2 amplitude and latency are also sensitive to *mental fatigue* (Boksem et al., 2005, 2006; Kato et al., 2009). In a study by Boksem et al. (2005) fatigue resulted in diminished N2 amplitude difference between relevant and irrelevant stimuli and in another study diminished difference between congruent and incongruent trials (Boksem et al., 2006) both indicative of impaired executive control. Others reported decreased N2 latency with time on task in a go/no-go task (Kato et al., 2009).

*P3 complex*. This complex consists of several components, the orientation-related P3a, the target-related P3b, and the no-go P3. P3a has a fronto-central amplitude maximum, while the amplitude maximum of P3b is at the centro-parietal locations (Squires et al., 1975; Comerchero and Polich, 1999; Polich, 2003). The no-go P3 has a frontal scalp distribution. Emergence of no-go P3 is interpreted as a correlate of response inhibition (Pfefferbaum et al., 1985). In *hypoxia* P3a amplitude diminished (Balázs et al., 2000) and in other studies the latency of this component increased (Fowler and Lindeis, 1992; Fowler and Prlic, 1995). Fowler and Prlic (1995) observed an inverted U function of P3 amplitude as a function of degree of hypoxia. Thakur et al. (2011) and Singh et al. (2004) found delayed P3b latencies. Ma et al. (2015) obtained decreased go and no-go P3 amplitude in the high-altitude group compared to the low-altitude group. As for other stress effects, Boksem et al. (2006) obtained increased P3b latency as a result of *mental fatigue*. Kato et al. (2009) also found a P3 latency augmentation for go and no-go trials, and P3 amplitude deterioration for no-go trials. Additionally, Massar et al. (2010) found decreased P3a amplitude after a cognitively demanding task. In *sleep deprivation* P3a decreased in an auditory oddball task (Gosselin et al., 2005) and in a modified ANT task P3b decreased (Trujillo et al., 2009) Additionally, Schapkin et al. (2006) obtained decreased no-go P3 amplitude after disturbed sleep.

Beyond the N2 and P3 components (likely connected to frontal functions), we also investigated the effect of extreme environment on early stages of information processing, reflected by the *N1/MMN* and *N1* components. There are several results showing that these components are also sensitive to stressors. For example, in a study by Yang et al. (2013) MMN amplitude decreased as a result of *mental fatigue* and in another study by Raz et al. (2001), MMN amplitude decreased after total *sleep* deprivation. In an ANT study by Trujillo et al. (2009), parietal N1 amplitude decreased after sleep deprivation.

## Materials and methods

### Subjects

A total of 13 male adults participated in the experiments (6 French, 6 Italians, and 1 British). ERP data from 3 participants and behavioral data from 2 participants had to be excluded from the data analysis because of one or more missing sessions in the ANT task and distraction data of one additional participant had to be excluded because of low performance.

The remaining 10/11 participants in the ANT task were 37.09/36.70 years old and the remaining 9/10 participants in the distraction task were 37.30/36.88 years old (20–55 years of age) and all reported normal hearing. Participants were members of the over-wintering crew at Concordia Antarctic Research Station in 2011. During their stay they performed these tasks on 7 occasions (Cycles) from mid-February to mid-November, once approximately every 6 weeks. ERP data from seventh session had to be excluded from the analysis because only a few subjects performed this session. Each subject participated in a 4-day-long fixed-sequence measurement at approximately every 6 weeks (SD 0.5). Supplementary Table 1 shows the measurement intervals. After some tests of other researchers, they performed our tasks on the second day. On each occasion, they performed the ANT task right after the distraction task. Participants performed the experiment in the same room under the same circumstances.

All subjects gave written informed consent. The study was approved by the Ethics Committee of the Department of Psychology of the University of Rome “La Sapienza.”

### Stimuli and procedure of distraction task

Subjects performed a sound duration discrimination task. For each tone presented, the subjects were asked to indicate by button presses whether it was short (200 ms) or long (400 ms). The frequency of sounds was either 440 Hz (86%, Standard) or 480 Hz (14%, Deviant). The task was composed of equal number of long and short tones to be responded to by pressing left/right mouse key. Assignment of left and right hand responses was counter-balanced between subjects. Participants were instructed to hold the mouse with both hands and operate it with two thumbs. Infrequent changes in frequency were irrelevant for the task; subjects were instructed to attend the duration information only.

The task comprised 4 stimulus blocks. In the first block (practice block), subjects were presented with 20 practice stimuli. The second block consisted of 150 non-practice stimuli and the third and fourth blocks consisted of 170 non-practice stimuli. All stimuli were presented binaurally via headphones (Sennheiser PMX 60). In the first block, subjects were given a “Correct” or “Incorrect” feedback displayed after each trial. SOA was random in the 1200–1400 ms range. Subjects were instructed to respond as quickly and accurately as possible.

### Stimuli and procedure of ANT task

**Figure 1:**
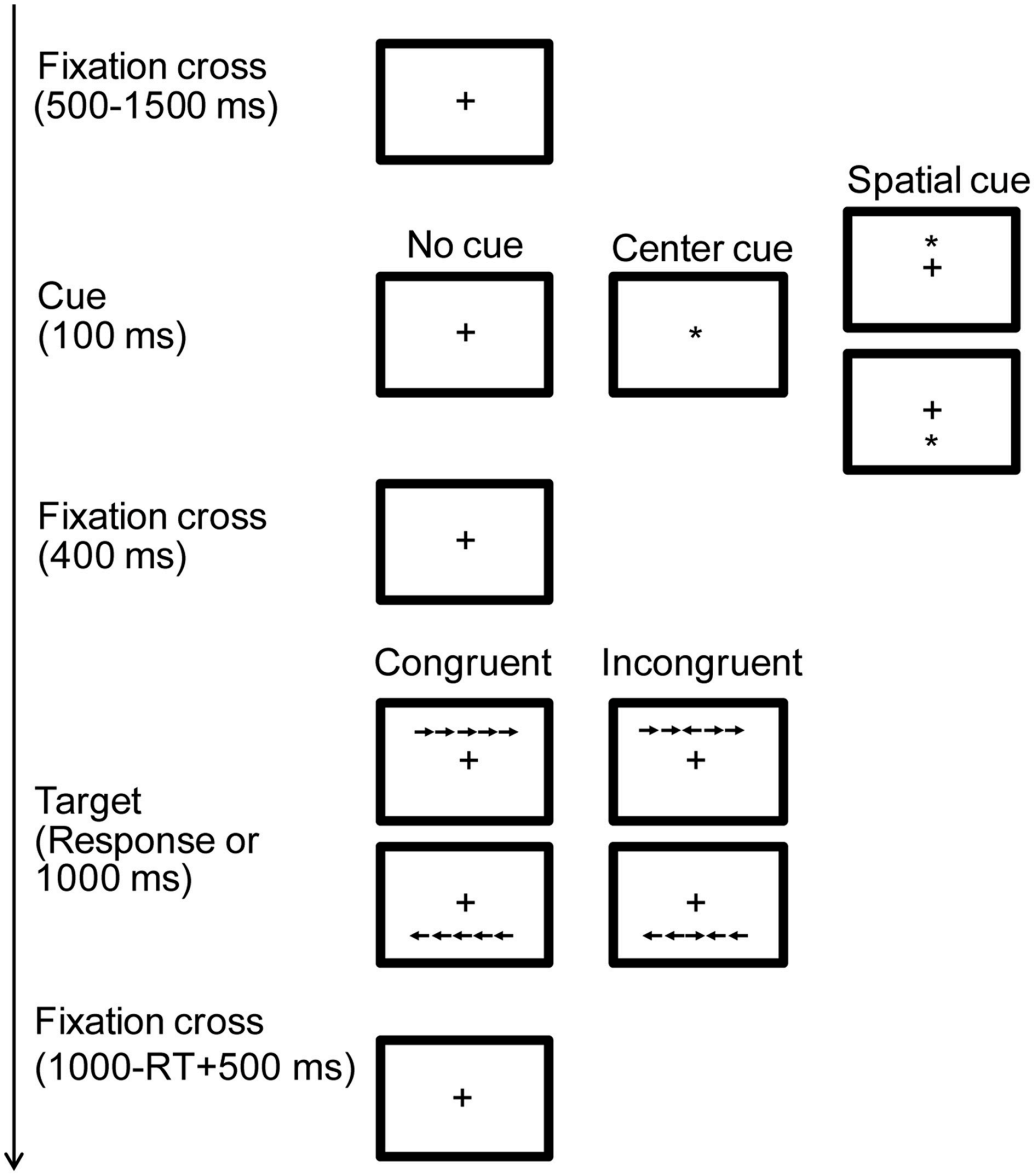
**Task design of Attention Network Test**. Each trial begins with a central fixation cross (+) followed by one of three cue types: No Cue, Center, or Spatial Cue. After the cue (^*^) five arrows appeared. The subjects had to press buttons according to the direction of the central arrow. The other four arrows (flankers) could point to the same (congruent condition) or to the opposite direction (incongruent condition). The arrows could arrive above or below the fixation cross. Timing information of the events is in the left side of the figure.

The test features a cued reaction time task. Figure 1 summarizes the stimuli and procedure. Each trial begins with a central fixation cross of variable duration (500–1500 ms). The fixation cross is followed by one of three cue types, that were equally likely to appear: No cue, Center or Spatial cue. Cues were presented for 100 ms, and consisted of an asterisk. Center cues appeared at the location of fixation cross, informing the subjects about the timing of the stimulus. Spatial cues appeared above or below the fixation cross and provided information about the timing as well as the location of the stimulus. A target display appeared 400 ms after the offset of the cue. The stimulus comprised five arrows. The subjects had to press buttons according to the direction of the central arrow. The other four arrows (flankers) could point to the same (congruent condition) or to the opposite direction (incongruent condition). The arrows could arrive above or below the fixation cross with equal probability. Each flanker type was equally likely. The target display remained on the screen until a response was made or 1000 ms elapsed.

The task comprised 240 trials divided in 3 blocks. Subjects were given an auditory feedback (beep) of incorrect responses during the entire task. Participants held the mouse with both hands and operated it with two thumbs. They were instructed to respond only to the direction of the central arrow in the target display as quickly and accurately as possible. Participants were told to maintain fixation at the fixation cross all the time.

### EEG recording and data analysis

EEG was recorded with BrainAmp amplifier and actiCap electrode cap (BrainProducts) using Fp1, Fp2, F7, F3, Fz, F4, F8, FC5, FC1, FC2, FC6, T7, C3, Cz, C4, T8, TP9, CP5, CP1, CP2, CP6, P7, P3, Pz, P4, P8, O1, Oz, O2 electrodes according to the extended 10–20 system referenced to FCz. AFz was used as ground. Data were online filtered at 0.5–70 Hz. Sampling rate was 250 Hz.

Extended ICA was performed on individual data sets to remove eye movement artifacts from EEG recordings (Delorme and Makeig, 2004; Onton and Makeig, 2006). Components representing eye blink and horizontal eye movement artifacts were identified by inspecting the component scalp map, time course and ERP-image (visualization of event-related signal variations across single trials) and were deleted. Eye movement-free EEG data were obtained by back-projecting the remaining ICA components to the time domain. EEG was low-passed filtered (20 Hz) and re-referenced to TP9 offline. Supplementary Image 1 shows the electrode locations.

### Data analysis of distraction task

Reaction times (RTs) were calculated as the median duration between stimulus onset and button press within the 150–1000 ms time range. Median RTs were computed separately for Standards and Deviants. Correspondingly, the overall percentages of correct responses were computed. To assess the effect of stimulus type and cycle on both RTs and accuracy, data were analyzed by two-factor [Stimulus Type (Deviant, Standard) × Cycle (1–6)] repeated measures ANOVA.

Epochs of 1200 ms were extracted (−100 to 1100 ms) and baseline corrected (−100 to 0 ms). Only trials with correct responses were analyzed. Epochs with the signal range exceeding 70 μV on frontal channels and 100 μV on non-frontal channels were discarded from the analyses. Grand-means were computed from the individual subject averages. Difference waves were formed by subtracting Standard ERPs from Deviant ERPs to evaluate the N1/MMN, P3a, and RON components. N1/MMN peak latency was identified as a negative peak at the Fz electrode site within the 130–300 ms range, P3a peak latency was identified as a positive peak at the Cz electrode site within the 250–500 ms range and RON peak latency was identified as a negative peak at the Fz electrode site within 450–750 ms range. Mean N1/MMN amplitude was measured as the average in a 40 ms time window, P3a and RON in 70 ms time windows centered at peak latencies. Mean amplitude values were calculated at the Fz, Cz and Pz electrode sites. The amplitude values were analyzed using Electrodes (Fz, Cz, Pz) × Cycle (1–6) repeated measures ANOVA.

### Data analysis of ANT task

RT and precision measures were calculated as above. Attention network effects were calculated as reaction time differences of the following conditions: alerting = RT_nocue_ − RT_centercue_; orienting = RT_centercue_ − RT_spatialcue_, inhibition = RT_incongruent_ − RT_congruent_.

To assess the effect of the target type, cue type and the cycle on RTs data were statistically evaluated by three-factor [Target Type (Incongruent, Congruent) × Cue Type (No Cue, Center Cue, Spatial Cue) × Cycle (1–6)] repeated measures ANOVA and three separate single-factor (6 Cycles) repeated measures ANOVAs, one for each attention network difference score (alerting, orienting and inhibition).

Epochs of 2000 ms were extracted (500 ms pre-cue to 1000 ms post-target including 500 ms cue-target interval) and baseline corrected (100–0 ms pre-cue). We identified 3 ERP components after the target stimuli: N1, N2, and P3. Trials were collapsed over all target conditions to analyze the effect of Cue type on N1. Target type (congruency) effects were analyzed by collapsing all cue conditions to identify N2 and P3.

N1 latency was identified at Oz electrode site within 120–280 ms range after the onset of target stimuli. N1 amplitude was collapsed across P7, P3, Pz, P4, P8, O1, Oz, and O2 electrode sites. Mean N1 amplitude was evaluated in a 40 ms time window centered at peak latency. The amplitude values of N1 were analyzed using Cue Type (No Cue, Center Cue, Spatial Cue) × Cycle (1–6) repeated measures ANOVA.

N2 component peak latency was identified as a negative peak at Fz electrode site within 200–380 ms after target stimuli. N2 amplitude was collapsed across FP1, FP2, F7, F3, Fz, F4, F8, FC5, FC1, FC2, FC6, C3, Cz, and C4. Mean N2 amplitude was evaluated in a 80 ms time window. The amplitude values were analyzed using Target Type (Incongruent, Congruent) × Cycle (1–6) repeated measures ANOVA.

This component exhibits a centro-parietal scalp distribution with a peak stimulus latency between 300 and 600 ms. Target-locked P3 component peak latencies were identified as a positive peak at Cz and Pz within the 300−600 ms range after the onset of target stimuli. Mean P3 amplitude was evaluated in a 100 ms time window centered at peak latencies. The mean amplitude values were measured at Fz, Cz and Pz. The amplitude values were analyzed using Electrode (Fz, Cz, Pz) × Cycle (1–6) × Target Type (Incongruent, Congruent) repeated measures ANOVA.

Greenhouse-Geisser correction was applied for all repeated measures with greater than 1 degree of freedom. Uncorrected degrees of freedom and corrected *p*-values are reported. Partial eta squared was computed as an estimate of effect size. Significant effects were further specified by Tukey-HSD *post-hoc* tests.

## Results

### Results of the distraction task

#### Behavioral performance

The Stimulus Type × Cycle ANOVA of reaction time showed a significant main effect of Stimulus Type [*F*_(1, 9)_ = 11.38, *p* < 0.01, $\eta_{\text{p}}^{2}$ = 0.55] and Cycle [*F*_(4, 55)_ = 3.37, *p* < 0.05, $\eta_{\text{p}}^{2}$ = 0.27]. RT for deviants was longer than RT for standards (*p* < 0.05). RT significantly decreased from cycle 1 to cycle 6 (significant pairs: cycle1–cycle6).

The Stimulus Type × Cycles ANOVA of the percentages of correct responses showed a significant main effect of Stimulus Type [*F*_(1, 9)_ = 8.32, *p* < 0.05, $\eta_{\text{p}}^{2}$ = 0.48]. Accuracy for Standards was better compared to Deviants (*p* < 0.05).

Table 1 summarizes the behavioral data.

**Table 1:** **Mean reaction time (ms) and hit rate for Deviant and Standard stimuli in each cycle (with standard error of mean)**.

**Cycle**	**1**.	**2**.	**3**.	**4**.	**5**.	**6**.
Reaction time for deviant (ms)	617.70 (7.17)	618.10 (14.16)	595.75 (10.02)	609.90 (10.61)	599.05 (15.84)	589.90 (13.79)
Hits for deviant (%)	85.42 (3.86)	88.57 (3.04)	88.88 (4.14)	88.71 (2.19)	90.8 (1.89)	90.57 (1.43)
Reaction time for standard (ms)	614.50 (11.12)	598.10 (11.77)	588.10 (11.32)	596.00 (9.72)	587.70 (14.72)	581.70 (11.81)
Hits for standard (%)	91.30 (1.65)	93.30 (1.40)	90.15 (3.28)	93.19 (1.48)	94.45 (1.28)	93.78 (1.19)

#### ERP results

##### N1/MMN

A significant main effect of Electrodes [*F*_(2, 16)_ = 7.87, *p* < 0.05, $\eta_{p}^{2}$ = 0.49] was obtained. N1/MMN amplitude was larger at Fz than Pz electrode site (*p* < 0.05).

##### P3a

A significant main effect of Electrodes [*F*_(2, 16)_ = 5.99, *p* < 0.05, $\eta_{p}^{2}$ = 0.42] was obtained. P3a amplitude was smaller at Pz compared to Cz electrode site (*p* < 0.05).

##### RON

A significant main effect of Electrodes [*F*_(2, 16)_ = 5.93, *p* < 0.05, $\eta_{p}^{2}$ = 0.42] was obtained. This component was larger at Fz than Pz site (*p* < 0.05).

**Figure 2:**
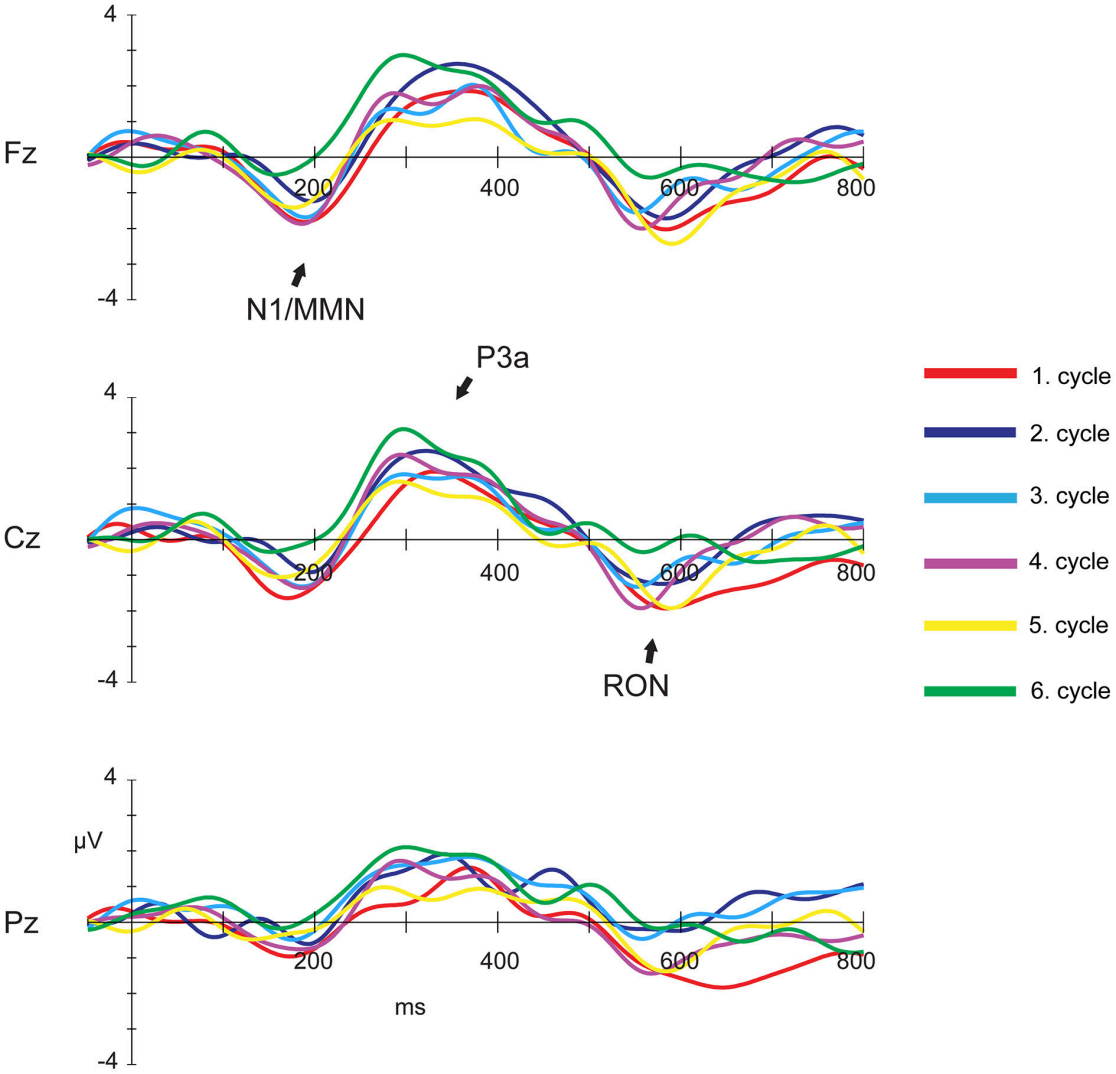
**Grand-mean difference waves (deviant ERPs–standard ERPs) for the six cycles depicted at electrode positions Fz, Cz, and Pz (filtered with a 10-Hz low-pass) in the distraction task**. In all cycles deviant stimuli elicited MMN, P3a and RON components.

**Figure 3:**
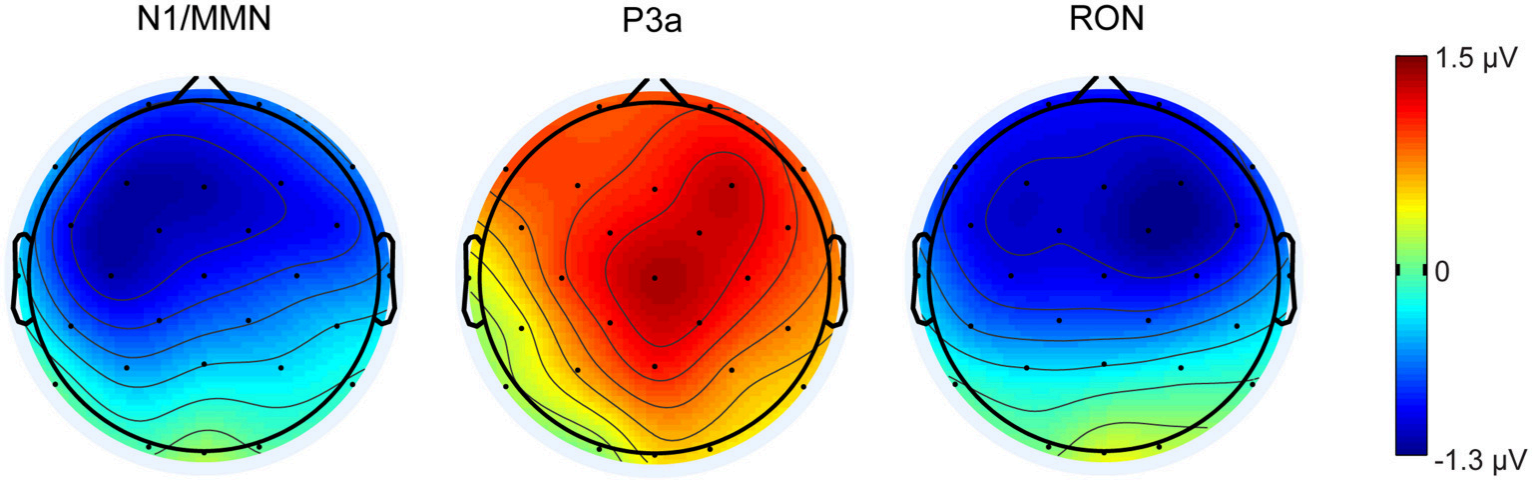
**Topographic distributions of the overall mean N1/MMN, P3a, and RON components (in μV) averaged across subjects and cycles presenting in 40 ms time window for MMN and in 70 ms time window for P3a and RON centered at peak latency**. Dots indicate electrode positions on the scalp.

Figure 2 illustrates the grand-mean deviant-minus-standard difference waveforms for the six cycles at Fz, Cz, and Pz electrode sites. Figure 3 demonstrates the topographic distribution of the overall mean N1/MMN, P3a, and RON components averaged across subjects and cycles. Supplementary Image 2 shows the grand-mean deviant-minus-standard difference waveforms for all electrodes for the six cycles.

### Results of the ANT task

#### Behavioral performance

For the RT data, a main effect of Cue Type [*F*_(2, 20)_ = 256.41, *p* < 0.001, $\eta_{\text{p}}^{2}$ = 0.96] and Target Type [*F*_(1, 10)_ = 839.89, *p* < 0.001, $\eta_{\text{p}}^{2}$ = 0.98], and an interaction between these two variables [*F*_(5, 50)_ = 2.63, *p* < 0.05, $\eta_{\text{p}}^{2}$ = 0.46] replicated earlier results (Fan et al., 2009). Participants were faster to correctly categorize targets in Spatial Cue trials than in No Cue or Center Cue trials (*p* < 0.001) and were also faster in Center Cue than No Cue trials (*p* < 0.001). RTs were longer to Incongruent than to Congruent targets (*p* < 0.001) and this effect was greatest for Center Cue.

A main effect of Cycle [*F*_(5, 50)_ = 9.78, *p* < 0.001, $\eta_{\text{p}}^{2}$ = 0.49] shows that RT decreased monotonically from cycle 1 until cycle 5 (significant pairs: cycle 1–cycle 3, 4, 5, and cycle 2–cycle 4). A marginally significant Target Type × Cycle [*F*_(5, 50)_ = 2.63, *p* = 0.08, $\eta_{\text{p}}^{2}$ = 0.20] and a significant Cue Type × Target Type × Cycle [*F*_(10, 100)_ = 3.55, *p* < 0.05, $\eta_{\text{p}}^{2}$ = 0.26] interactions were also obtained.

**Figure 4:**
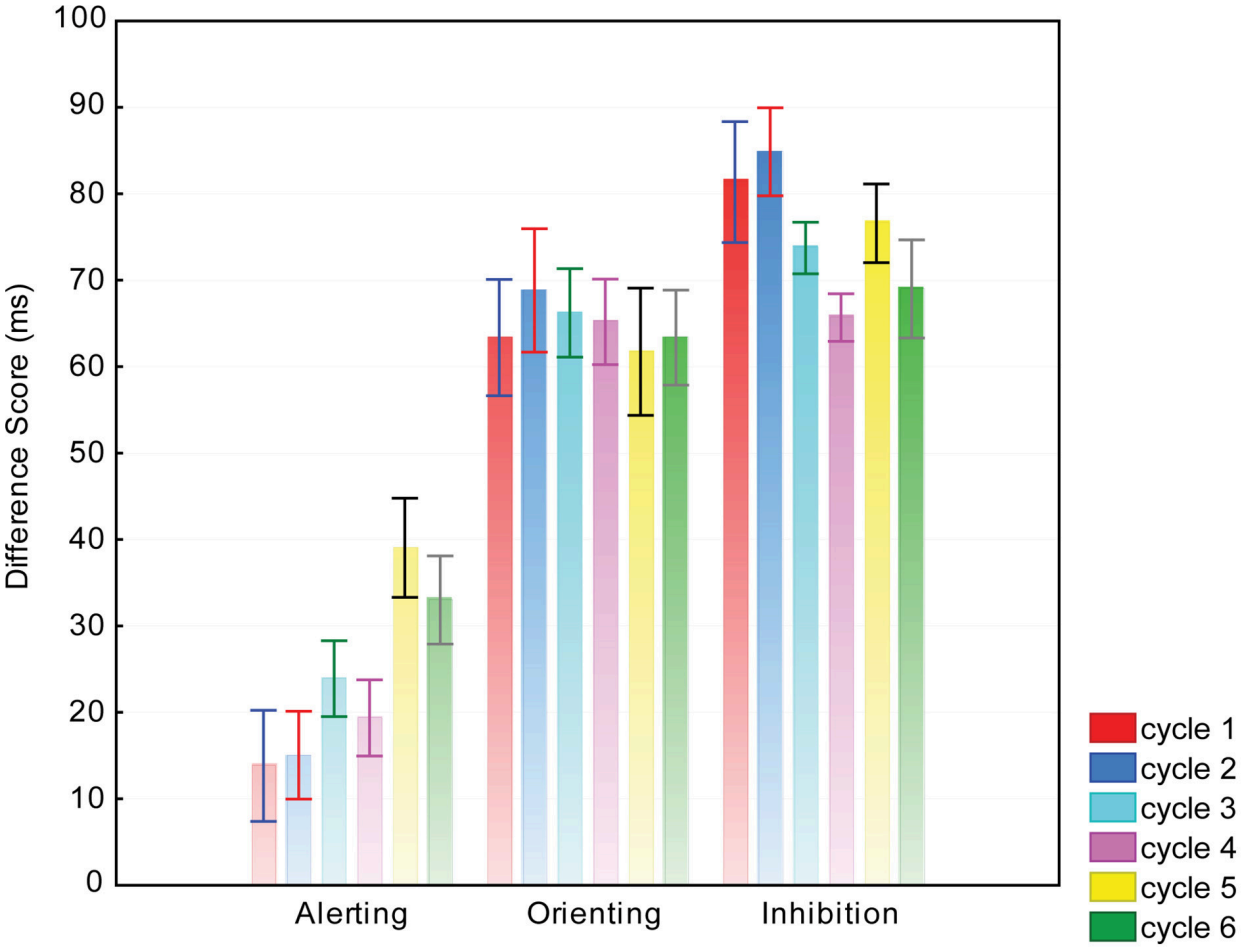
**Mean difference scores and standard errors for each of the three attention networks and six cycles**. Attention network effects were calculated as reaction time differences of the following conditions: alerting = RT_nocue_ − RT_centercue_; orienting = RT_centercue_ − RT_spatialcue_, inhibition = RT_incongruent_ − RT_congruent_.

Analysis of alerting difference scores demonstrated an effect of cycle [*F*_(5, 50)_ = 4.04, *p* < 0.05, $\eta_{\text{p}}^{2}$ = 0.28]. *Post-hoc* tests revealed an increasing alerting effect from cycle 1 until cycle 5 (significant pairs: cycle 1–cycle 5 and cycle 2–cycle 5). Analysis of inhibition difference scores demonstrated a marginal effect of cycle [*F*_(5, 50)_ = 2.63, *p* = 0.08, $\eta_{p}^{2}$ = 0.20]. A decreasing inhibition effect was found (significant pair: cycle 2–cycle 4). Figure 4 shows the mean difference scores and standard errors for each of the three attention networks and six cycle levels. Supplementary Table 2 summarizes the behavioral data.

#### ERP measures

##### N1

A significant main effect of Cue Type [*F*_(2, 18)_ = 7.15, *p* < 0.05, $\eta_{\text{p}}^{2}$ = 0.44] and a marginally significant main effect of Cycle [*F*_(5, 45)_ = 2.45, *p* = 0.07, $\eta_{\text{p}}^{2}$ = 0.21] were obtained. Smaller N1 component emerged for No Cue than for Center and Spatial Cue (*p* < 0.05). The amplitude of N1 was decreasing from cycle 1 to cycle 6 (significant pair: cycle 1–6).

**Figure 5:**
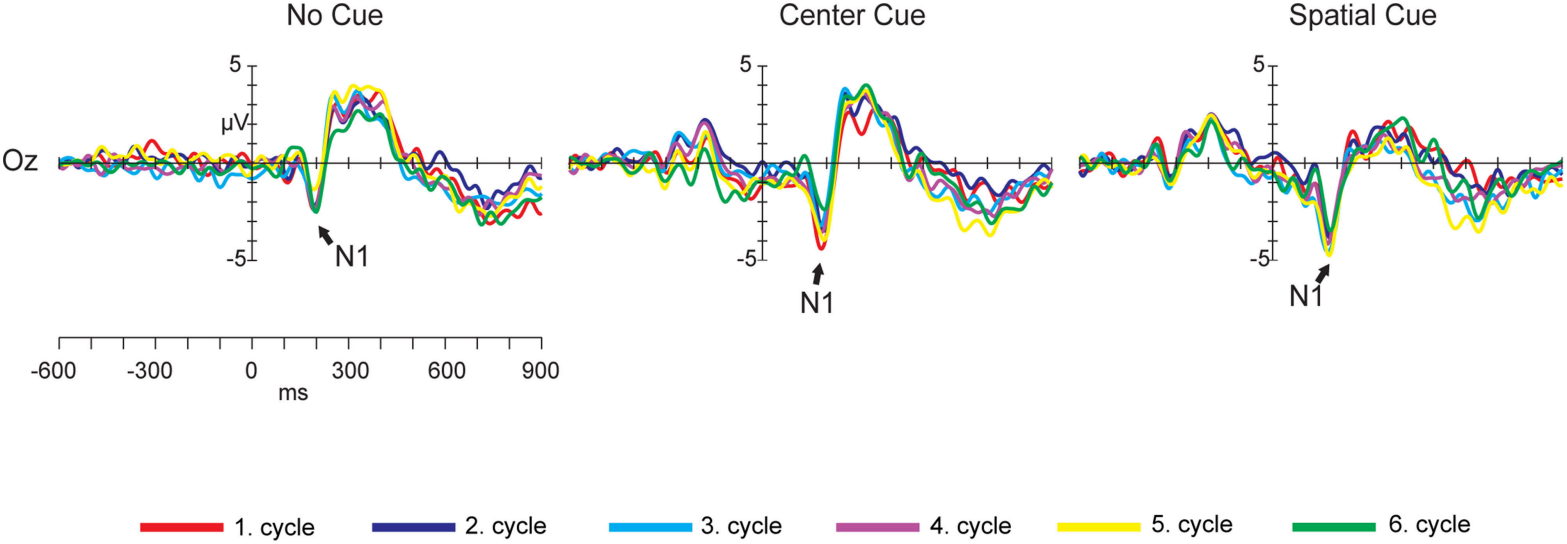
**Grand-mean event-related potential plots stratified by cue condition (No Cue, Center Cue and Spatial Cue) for Oz electrode site in the ANT task**. Arrows indicate the peaks of the N1 waveforms.

**Figure 6:**
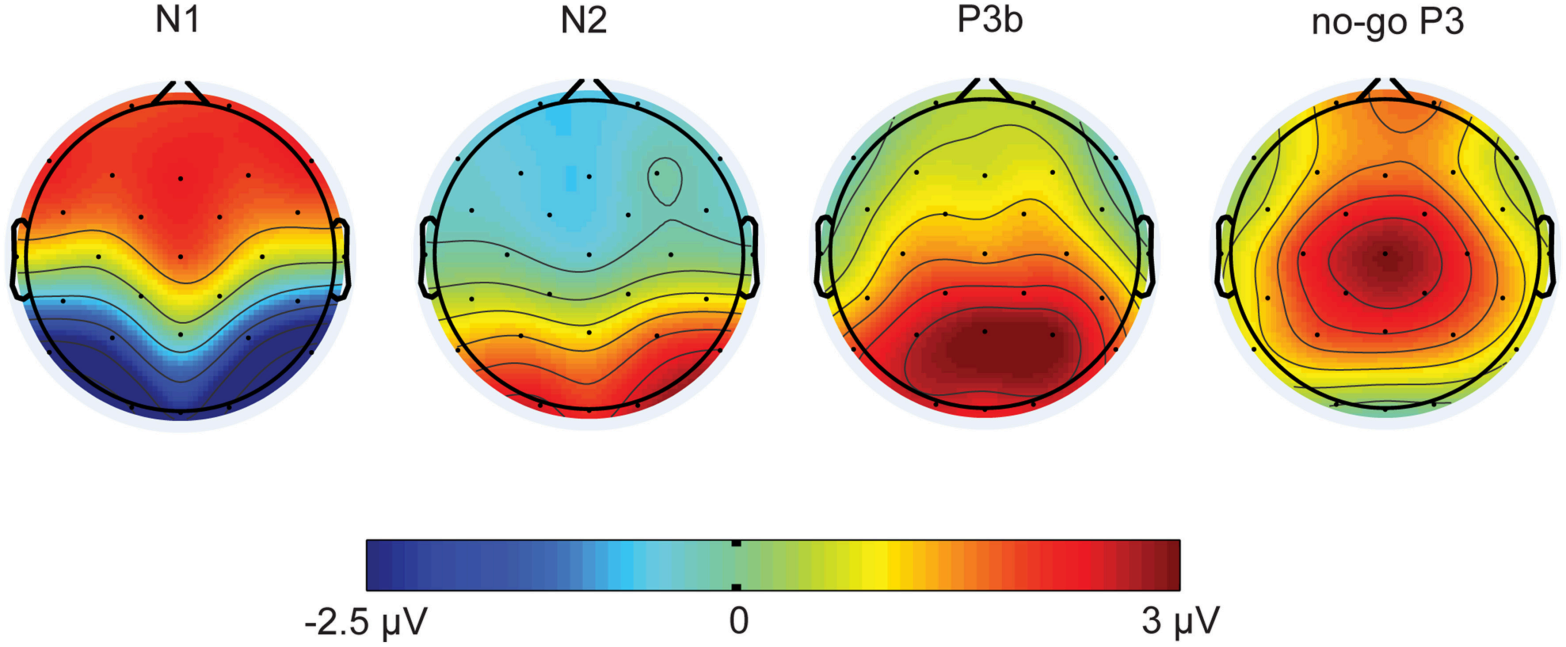
**Topographic distributions of the overall mean N1, N2, no-go P3, and P3b components (in μV) averaged across subjects and cycles presenting in 40 ms time window for N1, in 80 ms time window for N2 and 100 ms time window for no-go P3 and P3b centered at peak latency**. Dots indicate electrode positions on the scalp.

Figure 5 illustrates the grand-mean mean event-related potential plots stratified by cue condition (No Cue, Center Cue and Spatial Cue) for Oz electrode site. Figure 6 shows the topographic distributions of the overall mean N1 component (in μV) averaged across subjects and cycles presenting in 40 ms time window centered at peak latency.

Supplementary Images 3–5 show the grand-mean event-related potential plots stratified by cue condition for all electrodes for the six cycles.

##### N2

The Target Type × Cycle ANOVA did not show any significant effect. Congruent and Incongruent trials evoked similar N2 component.

**Figure 7:**
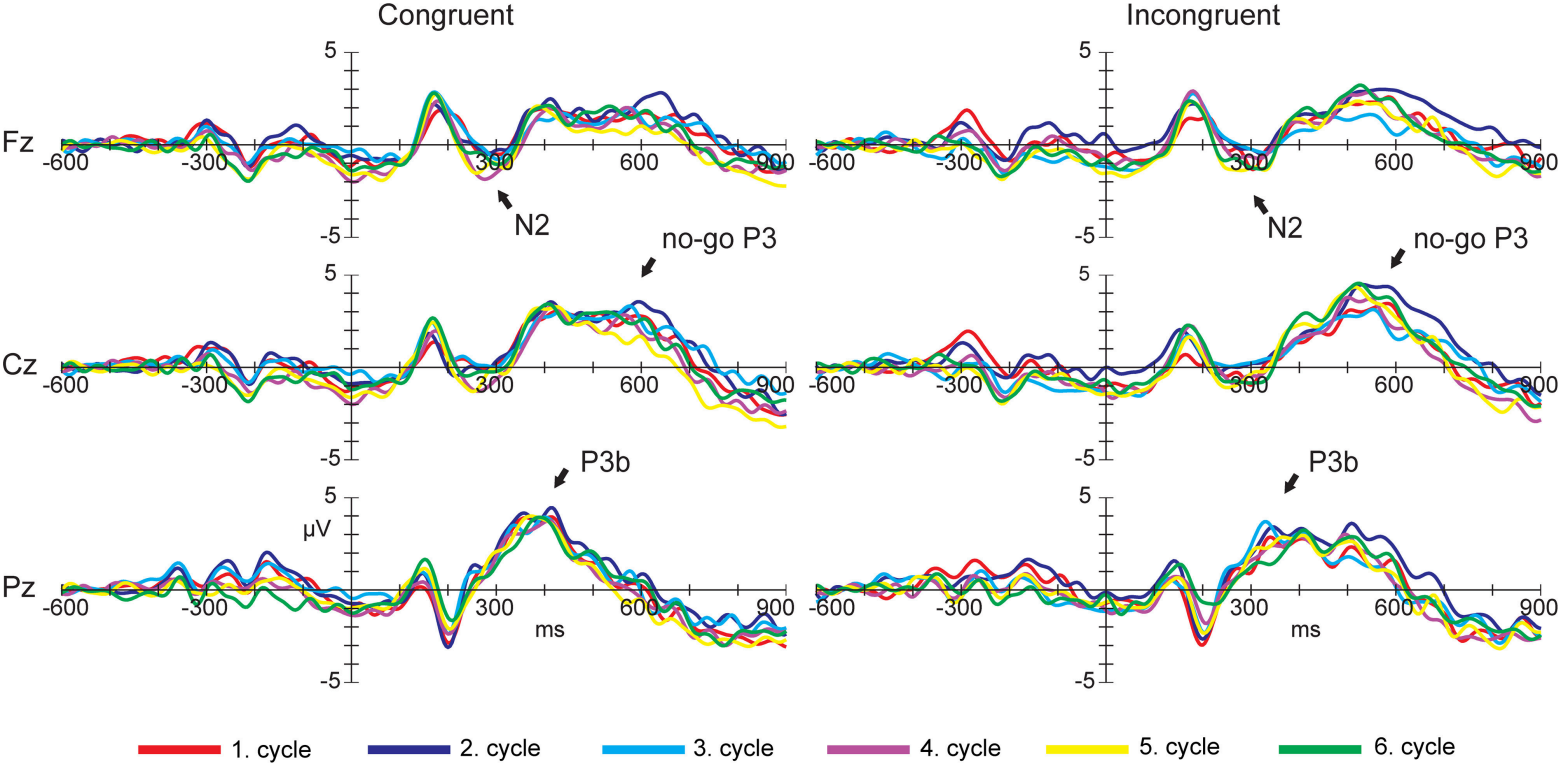
**Grand-mean event-related potential plots stratified by target condition (Congruent and Incongruent) for Fz, Cz, and Pz electrode sites in the ANT task**. Arrows indicate the peaks of the ERP waveforms.

Figure 7 illustrates the grand-mean event-related potential plots stratified by target condition (Congruent and Incongruent) for Fz, Cz, and Pz electrode sites.

Figure 6 shows the topographic distributions of the overall mean N2 component (in μV) averaged across subjects and cycles presenting in 80 ms time window centered at peak latency.

##### P3

A main effect of Target Type [*F*_(1, 9)_ = 12.00, *p* < 0.05, $\eta_{\text{p}}^{2}$ = 0.57], Electrode [*F*_(2, 18)_ = 13.53, *p* < 0.001, $\eta_{\text{p}}^{2}$ = 0.60], and a significant Target Type × Electrode interaction [*F*_(2, 18)_ = 11.50, *p* < 0.001, $\eta_{\text{p}}^{2}$ = 0.56] were obtained for the earlier P3 subcomponent, indicating a larger earlier P3 response for Congruent vs. Incongruent trials and smaller P3 response at Fz vs. Cz and Pz and Cz vs. Pz electrodes for Congruent and Incongruent trials. The difference between Congruent and Incongruent earlier P3 response was smaller at Fz compared to Cz and Pz electrode sites. The later peak of P3 was measured at Cz electrode. A main effect of Target Type [*F*_(1, 9)_ = 6.48, *p* < 0.05, $\eta_{\text{p}}^{2}$ = 0.41] and Electrode [*F*_(2, 18)_ = 10.31, *p* < 0.05, $\eta_{\text{p}}^{2}$ = 0.53] indicated that the later P3 response was smaller for Congruent vs. Incongruent trials and larger at Cz vs. Fz and Pz electrodes. No other main or interaction effects were significant for the early or late P3. Most importantly, there was no effect of Cycle.

Figure 7 illustrates the grand-mean event-related potential plots stratified by target conditions (Congruent and Incongruent) for Fz, Cz, and Pz electrode sites.

Figure 6 shows the topographic distributions of the overall mean no-go P3 and P3b components (in μV) averaged across subjects and cycles presenting in 100 ms time window centered at peak latency.

Supplementary Images 6, 7 show the grand-mean event-related potential plots stratified by target condition for all electrodes for the six cycles.

#### Diurnal Sp_O2_ values

Table 2 shows the mean diurnal Sp_O2_ values for each subject and 5 cycles. The Sp_O2_ values of cycle1 are missing.

**Table 2:** **Mean diurnal Sp_**O2**_ value for each subject and 5 cycles**.

**Subject**	**Cycle2**	**Cycle3**	**Cycle4**	**Cycle5**	**Cycle6**
1	89	90	94	96	89
2	96	90	96	93	94
3	94	90	92	93	89
4	91	88	95	94	88
5	88	93	94	93	86
6	90	86	86	91	90
7		91	95	98	96
8		89	95	95	94
9	88	88	94		88
10	89	88	93	93	88
11	88	88	89	92	87
12		87	88	93	86
13	87	88	94	95	87

*The Sp_O2_ values of cycle1 are missing*.

## Discussion

### Typical findings

Our results show typical effects both in the distraction task and in ANT task. In the distraction task reactions to Deviants were prolonged and less accurate compared to the reactions to Standards. Typical ERP responses to the task irrelevant changes were also obtained; N1/MMN, P3a, and RON were elicited for the deviating tones.

In the ANT task we replicated earlier results (Fan et al., 2009). The RT was smaller for Spatial Cue than for No Cue or Center Cue conditions and were also smaller for Center Cue than No Cue condition. RTs were faster for congruent than incongruent stimuli. Similarly to Neuhaus et al. (2010) and Galvao-Carmona et al. (2014), target-related N1 amplitude increased during alerting, and an amplitude increase of target-related N1 was found in Center Cue trials relative to No Cue trials. Contrary to the results of Neuhaus et al. (2010), no amplitude increase of target-related N1 was found in Spatial Cue trials relative to Center Cue. Our results do not support the idea of many other studies that spatial attention influences this component (Luck et al., 1994). The attention related later ERP components N2 and P3 in the ANT task show similar results to those observed by Neuhaus et al. (2010) and Neuhaus et al. (2007). No congruency effects were obtained on the N2 component, although we expected higher N2 amplitude for incongruent compared to congruent trials as this component relates to response inhibition or detection of conflict (Kok, 1986; Kiefer et al., 1998; Donkers and van Boxtel, 2004; Folstein and Van Petten, 2008; Smith et al., 2010). Wendt et al. (2007) actually found higher N2 amplitude to incongruent compared to congruent trials in an Eriksen flanker task.

We identified two target-locked P3 subcomponents, the earlier P3 response with parietal amplitude maximum and a later P3 response with central amplitude maximum (frontal in article of Neuhaus et al., 2010). We labeled the earlier subcomponent as P3b and the later subcomponent as no-go P3. P3b amplitude reduced and no-go P3 increased for the incongruent trials compared to congruent condition (similarly to Neuhaus et al., 2010). P3b reduction suggests increased decision uncertainty, because flanking arrows in incongruent trials induce ambiguity which is thought to decrease P3b amplitude (Johnson, 1986; Luck, 2005). On the other hand, larger no-go P3 amplitude presumably reflects inhibition of conflicting response tendencies in incongruent trials (Pfefferbaum et al., 1985).

### Results of repeated testing in antarctic conditions

The RTs were sensitive to the passage of time in both tasks. The decreasing RT in distraction and ANT tasks could be attributed to sustained effect of practice. Furthermore, two of the calculated attention network scores, specifically the alerting and inhibition effects also changed as a function of time. The alerting effect increased, which could indicate that participants better utilized central cues that alert them to trial onset. In accordance with a previous study (Ishigami and Klein, 2010) the inhibition effect decreased, which may derive from the better inhibition of the influence of surrounding flankers.

Contrary to our expectations the late ERP components (N2, P3, RON) related to frontal attentional functions did not show any significant changes.

As to the effect of time in the Antarctic environment on the early stage of information processing reflected by N1 and N1/MMN our study provided mixed results. Contrary to our expectations we only obtained a marginally significant decrease of N1 in the ANT task, but the N1/MMN component in the distraction task was not sensitive to the passage of time. Most likely the N1 amplitude reduction in the ANT task could be attributed to practice effect, as every other measure (RT and accuracy) shows that subjects improved constantly during their stay.

### Adaptation to antarctic environment

The lack of detectable cognitive deterioration might be attributed to the inappropriate sensitivity of the method, however, evidence shows that the cognitive functions which can be measured in these tasks are sensitive to stressors, such as *hypoxia* (Virués-Ortega et al., 2004), *mental fatigue* (Lorist et al., 2000), *sleep loss* (Jones and Harrison, 2001), and *impaired sleep quality* (Schapkin et al., 2006). Despite the limitations of the present study (the lack of baseline data collection before the expedition and the lack of control group), based on the results described above, it is more likely that the Antarctic conditions had no negative influence on the neurocognitive functions. Although the lack of detrimental effects seems clear from our results, the possible explanations for this result must be very cautious. As a tentative account, we summarize a few likely factors, that might have counteracted the negative influence of hypoxia, fluctuation of sunlight, isolation, confinement and other stressors.

First, several factors could influence crewmembers' sleep positively that might reduce the *sleep problems*. Crewmembers could take a nap in their free time. Research has shown that the amount of sleep per day is the most important factor of cognitive performance, irrespectively whether it is attained totally during the night or partly during the day in the form of napping (Mollicone et al., 2008). Moreover, they had the opportunity to exercise and according to Arendt (2005) sleep quality improves after physical activity. Indeed, Abeln et al. (2015) has shown that the mood of physically active crewmembers remained steady through the campaign compared to inactive.

Second, *slight deterioration of mood* that might affected some subjects (for example, physically inactive crewmembers, Abeln et al., 2015) does not necessarily lead to cognitive problems as previous studies have shown (Chepenik et al., 2007). Third, from a more psychological point of view, Antarctic missions also have positive aspects (Suedfeld, 2001, 2005) which counteract the impact of stressors. These *positive characteristics of the mission* are related on one hand to the environment (outside and inside, e.g., beauty of nature, comfortable and cozy habitat; Suedfeld, 2001; Atlis et al., 2004; Suedfeld, 2005). Moreover, crewmembers often experience personal growth (Palinkas, 1986; Kjærgaard et al., 2013) and enjoy social life (Suedfeld, 2001, 2005).

Furthermore, *humans are adaptive beings* and can adapt successfully to the negative attributes of a long-duration mission. In extreme situations people are able to manage stress and stay well (Antonovsky, 1987).

In acute hypoxia, especially when it is probed in laboratory settings devoid of other confounding factors as cold, fatigue and high level of physical exertion, the altitude of up to 3880 (+/− 500) m have not produced reliable cognitive deterioration in numerous studies (for no effect see Crow and Kelman, 1973; Paul and Fraser, 1994; Gustafsson et al., 1997; Takagi and Watanabe, 1999; Hewett et al., 2009). In longer sojourns, hypoxia attenuates with long-term acclimatization on various physiological levels, for example, red blood cell count increases in the first 5–6 weeks (Zubieta-Calleja et al., 2007).

Two studies reported results of the Antarctic overwintering at the Concordia station in 2011. In accordance with our results a study of Abeln et al. (2015) did not obtain any effect of Antarctic conditions on behavioral responses in cognitive tasks. Another study of Tellez et al. (2014) measured decreased nocturnal Sp_O2_ all through the campaign. Based on our results and those of Abeln et al. (2015) it seems that this mild systemic desaturation have not impaired brain oxygenation as compensatory processes (for example, red blood cell mass increase or cerebral vasodilation) effectively neutralized it.

The results of the present study might be regarded as additional evidence that it is possible to adapt to hypoxic conditions on long-term exposures, at least we have not detected any sign of detrimental effects by using tasks measuring mostly attentional functions.

Further research is needed to determine which factors play crucial role to counteract the stressors. Mapping these factors would be important in order to prevent deterioration of cognitive functions in future missions to extreme environments.

## Author contributions

IB, IC, LB: conception and design of the study; IB, ET: analyzed the data; IB, ET, IC, LB: drafting and/or revising the article; IB, ET, IC, LB: final approval.

## Funding

Life science research at Concordia Antarctic Station is supported by the European Space Agency (ESA), the Institute Polaire Paul Emile Victor (IPEV) and Programma Nazionale Ricerche in Antartide (PNRA). The work reported in this article was funded by ESA-PECS No. 4000103377.

### Conflict of interest statement

The authors declare that the research was conducted in the absence of any commercial or financial relationships that could be construed as a potential conflict of interest.
