# Supplementary material for: Extreme Environment Effects on Cognitive Functions: A Longitudinal Study in High Altitude in Antarctica
Source: Front Hum Neurosci. 2016 Jun 30;10:331. doi: 10.3389/fnhum.2016.00331 (PMC4928492; doi:10.3389/fnhum.2016.00331)
Supplement: Supplementary file 1 [file Table1.DOCX]

**Supplementary Table 1.**

Measurements intervals

| cycle1 | cycle2 | cycle3 | cycle4 | cycle5 | cycle6 |
| --- | --- | --- | --- | --- | --- |
| from mid-February to late-March | from late-March to early-May | from mid-May to mid-June | from mid-June to late-July | from early-August to mid-September | from mid-September to mid-October |
