# Supplementary figures and images for: Extreme Environment Effects on Cognitive Functions: A Longitudinal Study in High Altitude in Antarctica
Source: Front Hum Neurosci. 2016 Jun 30;10:331. doi: 10.3389/fnhum.2016.00331 (PMC4928492; doi:10.3389/fnhum.2016.00331)

**Supplementary Image 1.** Electrode locations.

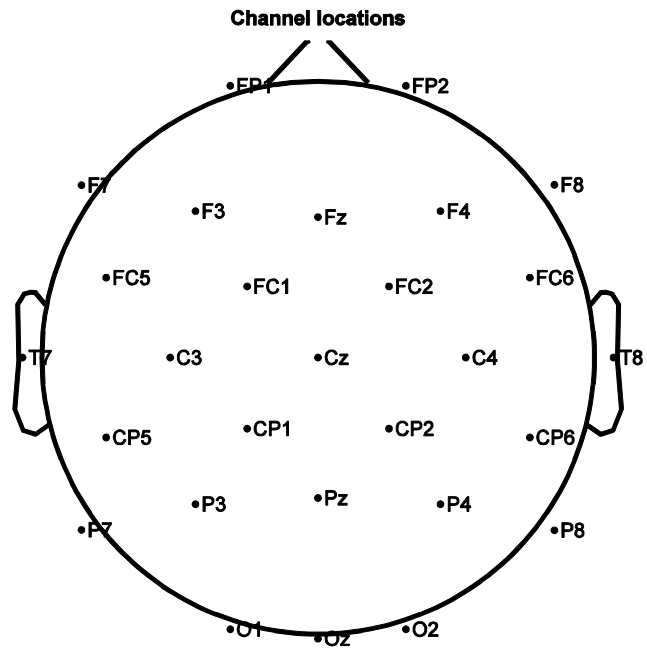

Supplement: Supplementary file 3 [file Image1.PDF]
