# Supplementary material for: Extreme Environment Effects on Cognitive Functions: A Longitudinal Study in High Altitude in Antarctica
Source: Front Hum Neurosci. 2016 Jun 30;10:331. doi: 10.3389/fnhum.2016.00331 (PMC4928492; doi:10.3389/fnhum.2016.00331)

Supplementary Image 3. Grand-mean event-related potential plots for No Cue condition for all electrodes in the ANT task. (1.cycle-red, 2.cycle-blue, 3.cycle-cyan, 4.cycle-purple, 5.cycle-yellow, 6.cycle-green).

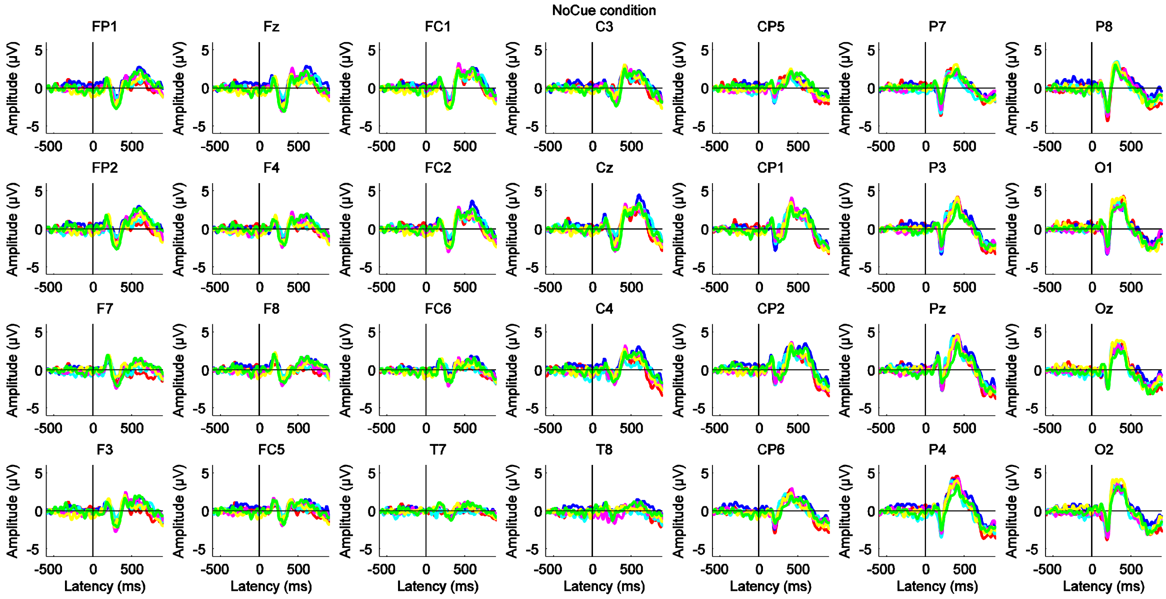

Supplement: Supplementary file 5 [file Image3.PDF]
